# Molecular Detection and Characterization of Zoonotic and Veterinary Pathogens in Ticks from Northeastern China

**DOI:** 10.3389/fmicb.2016.01913

**Published:** 2016-11-29

**Authors:** Feng Wei, Mingxin Song, Huanhuan Liu, Bo Wang, Shuchao Wang, Zedong Wang, Hongyu Ma, Zhongyu Li, Zheng Zeng, Jun Qian, Quan Liu

**Affiliations:** ^1^College of Life Science, Jilin Agricultural UniversityChangchun, China; ^2^Key Laboratory of Jilin Province for Zoonosis Prevention and Control, Military Veterinary Institute – Academy of Military Medical SciencesChangchun, China; ^3^College of Veterinary Medicine, Northeast Agricultural UniversityHarbin, China; ^4^Department of Pathology, The Second Clinical Medical School of Inner Mongolia University for the Nationalities, Inner Mongolia General Forestry HospitalYakeshi, China; ^5^Center for Prevention and Control of Animal Diseases of ChongqingChongqing, China

**Keywords:** tick-borne diseases, *Anaplasma*, *Ehrlichia*, *Babesia*, *Hepatozoon*, northeastern China

## Abstract

Tick-borne diseases are considered as emerging infectious diseases in humans and animals in China. In this study, *Ixodes persulcatus* (*n* = 1699), *Haemaphysalis concinna* (*n* = 412), *Haemaphysalis longicornis* (*n* = 390), *Dermacentor nuttalli* (*n* = 253), and *Dermacentor silvarum* (*n* = 204) ticks were collected by flagging from northeastern China, and detected for infection with *Anaplasma*, *Ehrlichia*, *Babesia*, and *Hepatozoon* spp. by using nested polymerase chain reaction assays and sequencing analysis. *Anaplasma phagocytophilum* was detected in all tick species, i.e., *I. persulcatus* (9.4%), *H. longicornis* (1.9%), *H. concinna* (6.5%), *D. nuttalli* (1.7%), and *D. silvarum* (2.3%); *Anaplasma bovis* was detected in *H. longicornis* (0.3%) and *H. concinna* (0.2%); *Ehrlichia muris* was detected in *I. persulcatus* (2.5%) and *H. concinna* (0.2%); *Candidatus* Neoehrlichia mikurensis was only detected in *I. persulcatus* (0.4%). The *Ehrlichia* variant (GenBank access number KU921424), closely related to *Ehrlichia ewingii*, was found in *H. longicornis* (0.8%) and *H. concinna* (0.2%). *I. persulcatus* was infected with *Babesia venatorum* (1.2%), *Babesia microti* (0.6%), and *Babesia divergens* (0.6%). Additionally, four *Babesia* sequence variants (GenBank access numbers 862303–862306) were detected in *I. persulcatus*, *H. longicornis*, and *H. concinna*, which belonged to the clusters formed by the parasites of dogs, sheep, and cattle (*B. gibsoni*, *B. motasi*, and *B. crassa*). Two *Hepatozoon* spp. (GenBank access numbers KX016028 and KX016029) associated with hepatozoonosis in Japanese martens were found in the collected ticks (0.1–3.1%). These findings showed the genetic variability of *Anaplasma*, *Ehrlichia*, *Babesia*, and *Hepatozoon* spp. circulating in ticks in northeastern China, highlighting the necessity for further research of these tick-associated pathogens and their role in human and animal diseases.

## Introduction

Ticks are second only to mosquitoes as vectors to transmit viral, bacterial, and protozoan agents in humans and animals, some of which pose a threat to human and animal health and are frequently zoonotic. Among tick-borne bacteria, members of the genera *Anaplasma* and *Ehrlichia* of the family Anaplasmataceae cause anaplasmoses and ehrlichioses in humans and animals (Ismail et al., 2010). The two most important species include *Ehrlichia chaffeensis*, the causative agent of human monocytic ehrlichiosis (HME), and *Anaplasma phagocytophilum*, the agent of human granulocytic anaplasmosis (HGA). Less commonly, ehrlichiosis induced by *Ehrlichia ewingii* was first discovered by Buller et al. (1999). Pritt et al. (2011) identified a third species of *Ehrlichia muris* in patients who had fever, malaise, headache, and lymphopenia in Wisconsin and Minnesota, the United States, and it has also been found in Europe and Asia (Rar et al., 2010; Tateno et al., 2015). Other species that infect animals include *A. marginale*, *Anaplasma centrale*, *A. ovis*, *E. canis*, and *Ehrlichia minasensis* (Wen et al., 2003; Kocan et al., 2015; Cabezas-Cruz et al., 2016). Several species have been described in China, such as *A. ovis*, *Anaplasma bovis*, and *A. platys* in Gansu province (Li et al., 2016), *A. centrale* and *E. canis* in Fujian province (Ge et al., 2016; Yu et al., 2016), and *E. chaffeensis* in Guangxi province (Yang et al., 2015b). In Asia, *Ixodes persulcatus* is considered the primary vector of *A. phagocytophilum* and *E. muris* (Jin et al., 2012; Ivanova et al., 2016).

The tick-borne protozoa of the genus *Babesia*, the causative pathogen of babesiosis in humans and animals, are considered as emerging diseases worldwide. Approximately 100 *Babesia* species have been identified to infect a broad range of animals, in which malaria-like disorders are induced (Rozej-Bielicka et al., 2015). Babesiosis has a great effect on the animal production and on companion animals; however, human babesiosis has attracted increased attention (Ord and Lobo, 2015). In immunocompetent individuals, the infection is usually asymptomatic, or shows mild, self-resolving symptoms, but babesiosis can be life-threatening in neonates/infants, elderly persons, asplenic patients, and the immunocompromised populations (Fang et al., 2015; Ord and Lobo, 2015). The three most important species to infect humans are *B. microti*, *B. divergens*, and *B. venatorum*. Other species, such as *B. ovis*, *B. major*, *B. bovis*, *B. bigemina*, *B. ovata*, *B. orientalis*, *B. motasi*, and *B. caballi*, cause animal infections (Fang et al., 2015). In China, *B. microti* has been found in rodents in Fujian, Zhejiang, Henan, and Heilongjiang provinces (Sun et al., 2008; Zhao et al., 2013; Chen et al., 2014); *B. divergens* has been described in *I. persulcatus*, *Haemaphysalis concinna*, and *H. japonica* and striped field mice in Heilongjiang province, where *B. venatorum* has also been reported in *I. persulcatus* (Fang et al., 2015; Jiang et al., 2015). Most ixodid tick species, such as *Ixodes scapularis* in the United States, *Ixodes ricinus* in Europe, and *I. persulcatus* in Asia, can transmit *Babesia* parasites to their natural hosts (Brasseur and Gorenflot, 1996; Sun et al., 2008; Schulze et al., 2013; Zamoto-Niikura et al., 2016).

Members of *Hepatozoon*, belonging to Apicomplexa protozoa, parasitize mainly erythrocytes in amphibians, reptiles, and avian hosts, whereas they are found primarily in leukocytes in mammals (Baneth, 2011). In humans, sporadic cases have been reported in Russia (Shuikina et al., 2004). In China, *Hepatozoon canis* has been reported in dogs from Beijing, Henan, Jiangsu, Shaanxi, and Xingjiang provinces (Xu et al., 2015). *Hepatozoon* DNA has been detected in *I. ricinus*, *Dermacentor* spp., and *Haemaphysalis* spp., but the vector competence remains to be confirmed (Giannelli et al., 2013; Hamsikova et al., 2016).

The topography of northeastern China includes both plains and mountains, where Changbai Mountains, Da Hinggan Mountains and Xiao Hinggan Mountains are not only the important natural barriers of protecting the ecosystem, but also host to a wide range of natural focal diseases, among which Lyme borreliosis and tick-borne encephalitis are the most common tick-borne diseases (Wu et al., 2013), and emerging tick-borne zoonoses, induced by *Rickettsia raoultii*, *Candidatus* Neoehrlichia mikurensis, and *B. venatorum*, have been reported (Fang et al., 2015). To the best of our knowledge, there is no wide survey of tick-borne pathogens in northeastern China. The objective of this study was to characterize tick-borne bacteria (*Anaplasma* and *Ehrlichia*) and protozoa (*Babesia* and *Hepatozoon*) in ticks collected from Jilin and Heilongjiang provinces, northeastern China. The data obtained here would contribute to understanding the epidemic and distribution of these tick-borne pathogens, which could be used to design effective control measures for tick-borne diseases in China.

## Materials and Methods

### Tick Collection and DNA Extraction

Questing adult ticks were collected by flagging vegetation in Jilin and Heilongjiang provinces, northeastern China, during April–May, 2015 (Liu et al., 2016). The obtained ticks were identified to species using the morphological method (Chen et al., 2010). In total, 253 *Dermacentor nuttalli*, 204 *Dermacentor silvarum*, 412 *H. concinna*, 390 *Haemaphysalis longicornis*, and 1699 *I. persulcatus* were collected in northeastern China, including 206 *D. nuttalli*, 175 *D. silvarum*, 244 *H. longicornis*, and 393 *I. persulcatus* from Jilin Province, and 47 *D. nuttalli*, 29 *D. silvarum*, 412 *H. concinna*, 146 *H. longicornis*, and 1276 *I. persulcatus* from Heilongjiang Province (Liu et al., 2016). The predominant tick species in northeastern China was *I. persulcatus* (58.0%), followed by *H. concinna* (14.0%), *H. longicornis* (13.0%), *D. nuttalli* (8.0%), *D. silvarum* (7.0%). The detailed information on the collected ticks is given in **Supplementary Figure S1**.

The ticks were pooled, approximately 15 female ticks per pool, according to their species and sampling site. Total DNA was extracted from crushed ticks using a TIANcombi DNA Lyse & Det PCR Kit (Tiangen Biotech, Co., Ltd, Beijing, China), and used to detect *Anaplasma*, *Ehrlichia*, *Babesia*, and *Hepatozoon* DNA by nested PCR assays.

### Polymerase Chain Reaction (PCR) Assays

The involved tick-borne pathogens were detected by nested PCR assays. The used primers were described in previous studies (Casati et al., 2006; Kawahara et al., 2006; Tabara et al., 2007; Cicuttin et al., 2014; Aydin et al., 2015; Sumrandee et al., 2015), or designed according to the conserved regions of target genes, as showed in **Table 1**.

**Table 1:** Oligonucleotide primers used for the detection of tick-borne pathogens.

Pathogen	Target gene	Oligonucleotide primer	Primer sequence (5′→3′)	Annealing temperature (°C)	Amplicon size (bp)	Reference
*Anaplasma*/*Ehrlichia* spp.	16S rRNA	EHR16SD	GGTACCYACAGAAGAAGTCC	52	345	Cicuttin et al., 2014
		EHR16SR	TAGCACTCATCGTTTACAGC			
*Anaplasma bovis*	16S rRNA	AB1F	CTCGTAGCTTGCTATGAGAAC	54	551	Kawahara et al., 2006
		AB1R	TCTCCCGGACTCCAGTCTG			
*Anaplasma phagocytophilum*	groEL	ANA-GroF	TCATTACTCAGAGTGCTTCTCAGTG	55	372	This study
		ANA-GroR	CGATCAAACTGCATACCATCAGTC			
*Ehrlichia* spp.	groEL	gro607F	GAAGATGCWGTWGGWTGTACKGC	55	730	Tabara et al., 2007
		gro1294R	AGMGCTTCWCCTTCWACRTCYTC			
		gro677F	ATTACTCAGAGTGCTTCTCARTG	58	364	
		gro1121R	TGCATACCRTCAGTYTTTTCAAC			
*Babesia* spp.	18S rRNA	BJ1-F1	GTCTTGTAATTGGAATGATGG	53	1123-1173	Casati et al., 2006
		BL-R1	GAATAATTCACCGGATCACTCG			This study
		BJ1-F2	GTCTTGTAATTGGAATGATGG	58	750-820	Casati et al., 2006
		BL-R2	ATTAACCAGACAAATCACTC			This study
*Hepatozoon* spp.	18S rRNA	HepF300	GCTAATACATGAGCAAAATCTCAA	54	1131	Sumrandee et al., 2015
		HepR900	CGGAA TTAA CCAGACAAAT			
		HepF	ATACATGAGCAAAATCTCAAC	59	643	Aydin et al., 2015
		HepR	CTTATTATTCCATGCTGCAG			

The reactions were conducted in an automatic thermocycler in a total volume of 25 μl containing 12.5 μl of Premix Taq (TaKaRa Taq Version 2.0 plus dye), 0.5 μl of each primer (5 pmol), 2.0 μl of template DNA (∼60 ng), and 9.5 μl of distilled water. The reaction conditions of the first-round amplification included 5 min of pre-denaturation at 94°C; followed by 30 cycles at 94°C for 30 s, annealing for 30 s at an appropriate temperature, and 72°C for 1 min; with a final extension at 72°C for 10 min. During the second round of amplification, 1 μl of the product from the first-round amplification was used as the template to amplify the target genes using the nested primers; the amplification included 30 cycles of 30 s at 94°C, annealing for 30 s at an appropriate temperature, and 40 s at 72°C.

For *Anaplasma*/*Ehrlichia* detection, a 345-bp fragment of the 16S rRNA was amplified using the universal primers EHR16SD and EHR16SR. The *Anaplasma*-positive samples could be identified to species by PCR using the species-specific primers, and the *Ehrlichia*-positive samples were further identified to species by the nested PCR and sequencing.

### Phylogenetic Analysis

The PCR products were purified and sequenced in both directions using the specific primers. Nucleotide sequences were analyzed by BlastN and aligned with ClustalW. The pairwise distance was analyzed using the Kimura’s 2-parameter model. Phylogenetic analyses were conducted using the software MEGA 5^1^. The neighbor-joining method was employed to construct a phylogenetic tree. The reliability of branches in the tree was evaluated by bootstraping analysis with 1000 replicates, and the bootstrap value more than 60% was considered significant.

### Statistical Analysis

The prevalence of infection in ticks was calculated using the program PooledInfRate version 4.0 (Biggerstaff, 2009). Statistical analyses were performed using SPSS version 17.0, SPSS, Inc., Chicago, IL, USA. *p*-value less than 0.05 was considered statistically significant.

## Results

### *Anaplasma* DNA in Ticks

**FIGURE 1:**
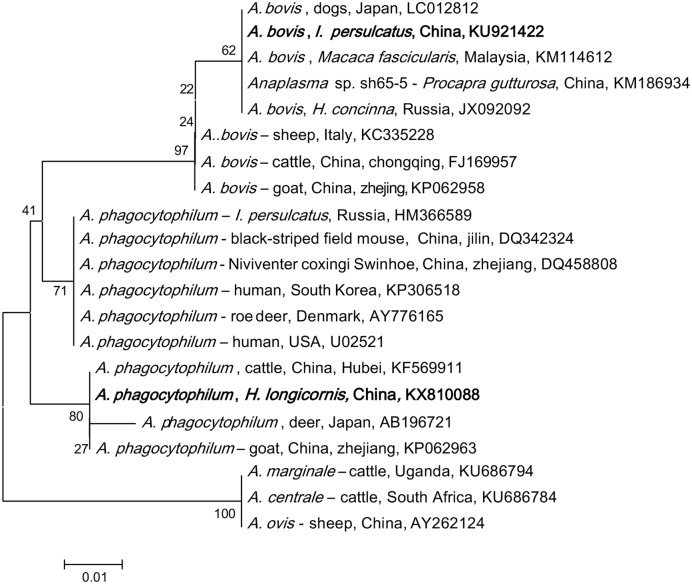
**Phylogenetic analysis of the partial 16S rRNA gene (301 bp) from *Anaplasma* spp. in ticks of northeastern China.** The phylogenetic trees were constructed by the Neighbor-Joining method using the Kimura’s 2- parameter model. A total of 300 positions were included in the final analysis. Sequences are identified by their strain name and the origin, followed by the GenBank accession number. The detected *Anaplasma* of the present study is marked in bold. The scale bars in each panel indicate 0.01 substitutions per site.

*Anaplasma* DNA was detected in *I. persulcatus*, *H. concinna*, *H. longicornis*, *D. silvarum*, and *D. nuttalli*. Phylogenetic analysis of the partial 16S rRNA gene showed that the detected *Anaplasma* belonged to *A. phagocytophilum* and *A. bovis* (**Figure 1**). *A. phagocytophilum* in this study was phylogenetically clustered together with those found in goat from Zhejiang (KP062963), cattle from Hubei in China (KF569911), and deer in Japan (AB196721), which formed a unique haplotype (100% identity), and was distinct from the other haplotypes, including the isolates in human from USA (U02521), Denmark (AY776165), South Korea (KP306518), and Russia (HM366589; **Figure 1**; Supplementary Table S1). All the obtained *A. bovis* 16S rRNA gene sequences were 100% identical to that found in dogs in Japan (LC012812), *Macaca fascicularis* in Malaysia (KM114612), and *H. concinna* in Russia (JX092092), which formed a haplotype different from that detected in cattle in Chongqing (FJ169957), and goat in Zhejiang (KP062958) in China, and sheep in Italy (KC335228; **Figure 1**).

*Anaplasma phagocytophilum* was found in all tick species in both provinces, with a higher prevalence in Heilongjiang (4.5% in Jilin and 7.2% in Heilongjiang, *p* < 0.05, Supplementary Table S2). High prevalence was detected in *I. persulcatus* (9.4%, *p* < 0.05) as comparison with that in *D. silvarum* (2.3%), *H. concinna* (1.9%), and *D. nuttalli* (1.7%), suggesting that *I. persulcatus* may be the primary vector for *A. phagocytophilum* (**Table 2**). *A. bovis* was only found in *H. longicornis* (0.7%) and *H. concinna* (0.2%) collected from Heilongjing province, without significant difference between the two species (*p* > 0.05, Supplementary Table S1).

**Table 2:** Molecular detection of zoonotic and veterinary pathogens in ticks from northeastern China.

Pathogen genera	Pathogen species (GenBank access number)^a^	*Ixodes persulcatus* (%, 95% CI)	*Haemaphysalis longicornis* (%, 95% CI)	*Haemaphysalis concinna* (%, 95% CI)	*Dermacentor nuttalli* (%, 95% CI)	*Dermacentor silvarum* (%, 95% CI)
*Anaplasma*	*Anaplasma phagocytophilum* (KX810088)	9.4 (7.5–11.7)^b,c,d^	1.9 (0.9–3.7)^b,e^	6.5 (4.0–10.3)^e,f^	1.7 (0.6–4.0)^c,f^	2.3 (0.8–5.7)^d^
	*Anaplasma bovis* (KU921422)	0	0.3 (0.1–1.2)	0.2 (0.1–1.2)	0	0
*Ehrlichia*	*Ehrlichia muris* (KU921423)	2.5 (1.8–3.4)^b^	0	0.2 (0.1–1.2)^b^	0	0
	*Candidatus* Neoehrlichia mikurensis (KU921420)	0.4 (0.2–0.9)	0	0	0	0
	*Ehrlichia* sp.hc-hlj209 (KU921424)	0	0.8 (0.2–2.1)	0.2 (0.1–1.2)	0	0
*Babesia*	*Babesia venatorum* (KU862302)	1.2 (0.8–1.9)	0	0	0	0
	*Babesia microti* (KU862301)	0.6 (0.3–1.0)	0	0	0	0
	*Babesia divergens* (KU862301)	0.1 (0.1–0.4)	0.3 (0.1–1.2)	0	0	0
	*Babeisia* sp.hc-hlj212 (KU862304)	0	0	0.2 (0.1–1.2)	0	0
	*Babesia* sp.Ip-hlj179 (KU862305)	0.1 (0.0–0.3)	0	1.0 (0.3–2.5)	0	0
	*Babesia* sp.hl-hlj178 (KU862306)	0	0.3 (0.1–0.2)	0.8 (0.2–2.1)	0	0
	*Babesia* sp.Ip-hlj238 (KU862303)	0.1 (0.0–0.3)	0	0.5 (0.1–1.6)	0	0
*Hepatozoon*	*Hepatozoon* sp. hlj-Ip229 (KX016028)	0.1 (0.0–0.3)	0	0.5 (0.1–1.6)	0.8 (0.1–2.6)	0
	*Hepatozoon* sp. hlj-dn242 (KX016029)	1.8 (1.2–2.6)	1.0 (0.4-2.4)	0	0.8 (0.1–2.6)	3.1 (1.2–6.9)

^a^*Ehrlichia sp.hc-hlj209 (KU921424) detected in H. concinna collected in Heilongjiang; Babesia sp. hc-hlj212 (KU862304) detected in H. concinna; Babesia sp.Ip-hlj179 (KU862305) detected in I. persulcatus; Babesia sp.hl-hlj178 detected in H. longicornis; Babesia sp.Ip-hlj238 (KU862303) detected in I. persulcatus; Hepatozoon sp. hlj-Ip229 detected in I. persulcatus in Heilongjiang province; Hepatozoon sp. hlj-dn242 detected in D. nuttalli in Heilongjiang province.*

^b,c,d,e,f^*Significant difference was found between the prevalence in the two tick species (p < 0.05), analyzed by the Fisher’s exact test.*

### *Ehrlichia*-Specific DNA in Ticks

**FIGURE 2:**
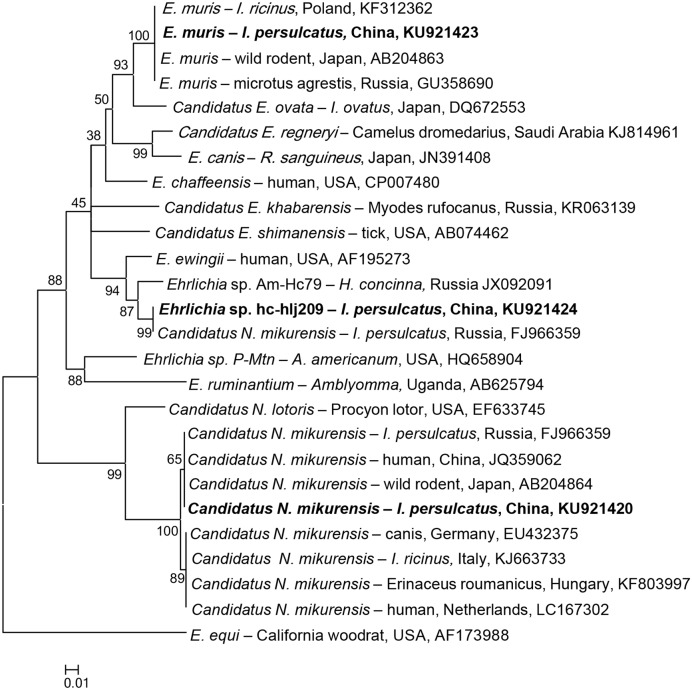
**Phylogenetic analysis of the partial groEL gene (287 bp) from *Ehrlichia* spp. in ticks from northeastern China.** The phylogenetic trees were constructed by the Neighbor-Joining method using the Kimura’s 2- parameter model. A total of 284 positions were included in the final analysis. Sequences are identified by their strain name and the origin, followed by the GenBank accession number. The detected *Ehrlichia* of the present study is marked in bold. The scale bars in each panel indicate 0.01 substitutions per site.

*Ehrlichia*-specific DNA was detected in *I. persulcatus*, *H. longicornis*, and *H. concinna*, and the phylogenetic analysis of the partial heat shock protein (groEL) gene showed that the detected sequences were grouped with *E. muris*, *Candidatus* N. mikurensis, and a possible novel variant (**Figure 2**).

All the obtained *E. muris* groEL gene sequences were 100% identical to those of *E. muris* detected in *I. ricinus* from Poland (KF312362), *I. persulcatus* from Russia (GU358686), and *Microtus agrestis* from Russia (GU358690, Supplementary Table S3). *E. muris* was tested in *I. persulcatus* (1.9%) and *H. concinna* (0.2%) in Heilongjiang, and *I. persulcatus* (4.3%) in Jilin, with significant difference of infection rate in *I. persulcatus* between the two provinces (*p* < 0.05, Supplementary Table S4). The overall infection rate for *E. muris* was 2.5% in *I. persulcatus*, significantly higher than that in *H. concinna* (0.2%) in northeastern China (*p* < 0.05, **Table 2**).

The detected *Candidatus* N. mikurensis groEL sequences were 100% identical to the *Candidatus* N. mikurensis sequences detected in *I. persulcatus* from Russia (FJ966359) and in wild rodents of Japan (AB204864), and humans from China (JQ359062, Supplementary Table S3), which were phylogenetically clustered, distinctive from the European countries, including Hungary, Germany, Netherlands, and Italy (**Figure 2**). *Candidatus* N. mikurensis were only found in *I. persulcatus* in Jilin (0.3%) and Heilongjiang (0.5%), and no significant difference was found between the two provinces (*p* > 0.05, Supplementary Table S4).

The typical variant, *Ehrlichia* sp. Kh-Hj27 found in Russia, which showed the highest similarity (96%) to that of *E. ewingii* (AF195273, Supplementary Table S3), was also detected in both *H. longicornis* (2.2%) and *H. concinna* (0.2%) in Heilongjiang province, clustered together with one more *Ehrlichia* genetic variant (Am-Hc79, JX092091) detected in *H. concinna* in Russia (97% identity), thus forming a separate branch on the phylogenetic tree (Supplementary Table S3; **Figure 2**).

### *Babesia* DNA in Ticks

**FIGURE 3:**
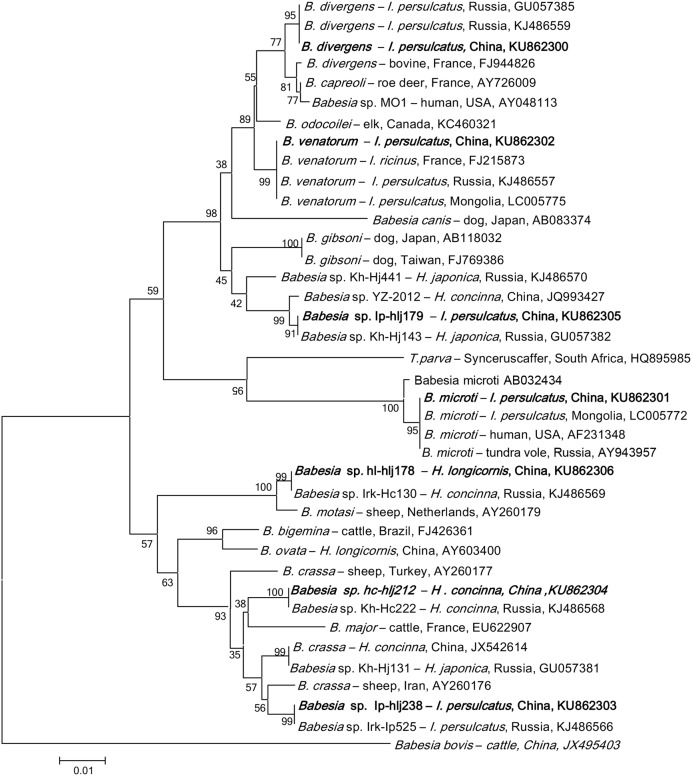
**Phylogenetic analysis of the partial 18S rRNA gene (740 bp) from *Babesia* spp. in ticks from northeastern China.** The phylogenetic trees were constructed by the Neighbor-Joining method using the Kimura’s 2- parameter model. A total of 721 positions were included in the final analysis. Sequences are identified by their strain name and the origin, followed by the GenBank accession number. The detected *Babesia* of the present study is marked in bold. The scale bars in each panel indicate 0.01 substitutions per site.

*Babesia* DNA was detected in *I. persulcatus*, *H. longicornis*, and *H. concinna* in Jilin and Heilongjiang provinces of northeastern China, but positive results was obtained neither from *D. nuttalli* nor from *D. silvarum*. Phylogenetic analysis showed that the *Babesia* species in ticks from Heilongjiang and Jilin provinces were clustered together with *B. venatorum*, *B. microti*, *B. divergens*, and the four *Babesia* genetic variants belonged to the carnivores, cattle and small ruminants groups (**Figure 3**), with the DNA sequence homology of 99–100% (GenBank accession numbers: KU862300–KU862306).

The obtained gene sequences of *B. venatorum* 18S rRNA were 100% identical to each other and to those found in *I. ricinus* of France (FJ215873), and in *I. persulcatus* of Russia (KJ486557) and Mongolia (LC005775, Supplementary Table S5), and the phylogenetic analysis showed that the *B. venatorum* from Jilin and Heilongjiang provinces of China and Europe clustered in the same clade, but distinct from other *Babesia* species (**Figure 3**). Only *I. persulcatus* (1.2%) was detected to be infected with *B. venatorum*, with a prevalence of 0.3% in Jilin and 1.6% in Heilongjiang (*p* < 0.05, **Table 2**; Supplementary Table S6). Other ticks, including *Dermacentor* and *Haemaphysalis*, were detected negative.

Nine *I. persulcatus* pools were detected positive for *B. microti*, whose sequences completely matched the *B. microti* strains isolated from humans in the United States (AF231348), tundra vole of Russia (AY943957), and from *I. persulcatus* of Mongolia (LC005772, Supplementary Table S5), the phylogenetic analysis indicated that these isolates were clustered in the same clade (**Figure 3**). The overall prevalence of *B. microti* was 0.6% in *I. persulcatus*, with a prevalence of 1.1% in Jilin and 0.4% in Heilongjiang (*p* > 0.05, Supplementary Table S6). The other tick species were tested negative. Despite the high similarity of *B. microti* found in northeastern China to highly pathogenic strains, human babesiosis caused by *B. microti* has not been confirmed in northern China to date. We cannot, however, rule out the existence of human cases.

Two *H. longicornis* pools and one *I. persulcatus* pool in Heilongjiang province were tested positive for *B. divergens* variant, showing a prevalence of 0.7 and 0.3%, respectively, (Supplementary Table S6). The obtained 18S rRNA gene sequences were 100% identical to the strains isolated from *I. persulcatus* in Russia (GU057385 and KJ486559, Supplementary Table S5), but differed from both the European *B. divergens* and *B. capreoli* isolates, and the *Babesia* sp. MO1 isolate recovered from humans in the United States, and formed a separate clade (**Figure 3**). Thus, we cannot group this genetic variant with any particular species.

In addition, four *Babesia* sequence variants were detected in *H. longicornis*, *H. concinna*, and *I. persulcatus* (**Table 2**), which were closely related to the groups of carnivores, cattle and small ruminant *Babesia* (**Figure 3**). These variants have also been found in Russia (GU057382, KJ486566, KJ486568, and KJ486569), with the 18S rRNA gene sequences of 100% identify (Supplementary Table S5), but their mammalian host species remain unknown (Rar et al., 2011).

### *Hepatozoon* DNA in Ticks

**FIGURE 4:**
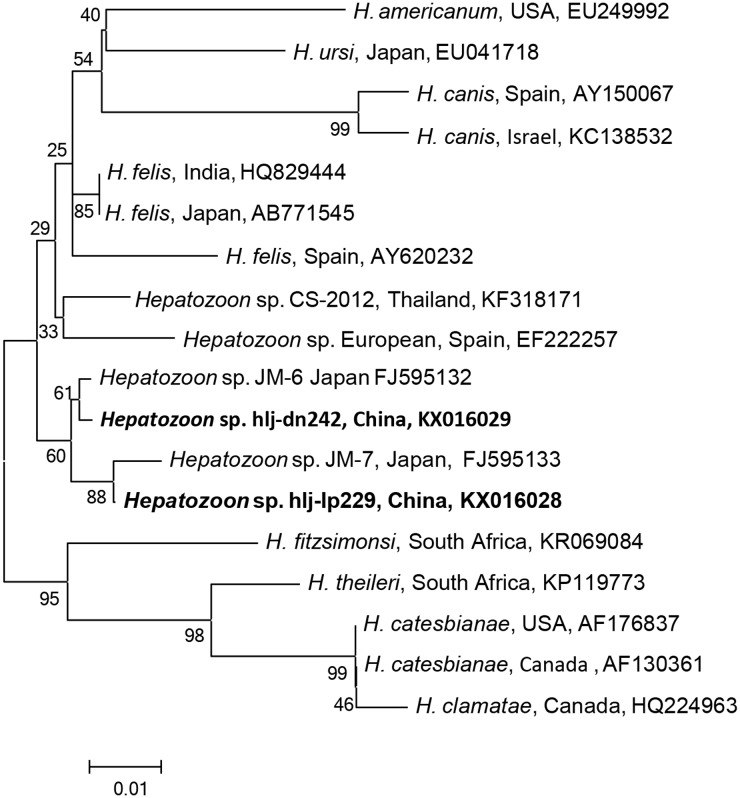
**Phylogenetic analysis of the partial 18S rRNA gene (343 bp) from *Hepatozoon* spp. in ticks from northeastern China.** The phylogenetic trees were constructed by the Neighbor-Joining method using the Kimura’s 2- parameter model. A total of 341 positions were included in the final analysis. Sequences are identified by their strain name and the origin, followed by the GenBank accession number. The detected *Hepatozoon* of the present study is marked in bold. The scale bars in each panel indicate 0.01 substitutions per site.

The *Hepatozoon* DNA was detected in *D. nuttalli*, *D. silvarum*, *H. concinna*, *H. longicornis*, and *I. persulcatus*, which were phylogenetically divided into two groups (**Figure 4**), and were identical (99–100%) to *Hepatozoon* isolates JM-6 (FJ595132), and JM-7 (FJ595133) (Supplementary Table S7), isolated from Japanese martens (*Martes melampus melampus*) (Kubo et al., 2009). The prevalences of *Hepatozoon* sp. were very low in all tick species, ranging from 0.1 to 4.4%, and no significant difference was found (*p* > 0.05, **Table 2**, Supplementary Table S8). No other *Hepatozoon* species was detected in ticks from Jilin and Heilongjiang provinces of Northeastern China.

## Discussion

Several species of *Anaplasma*, including *A. phagocytophilum*, *A. bovis*, *A. marginale*, and *A. ovis*, have been described in China (Li et al., 2016; Zhang et al., 2016). *A. phagocytophilum* is considered as an emerging human pathogen of public health importance, which is naturally maintained in tick-mammal cycles, and has been found in sheep, goats, cattle, rabbits, and rodents (Jin et al., 2012). Although, *A. phagocytophilum* was detected in the genera of *Ixodes*, *Dermacentor*, and *Haemaphysalis*, *I. persulcatus* may play the most important role in the transmission of the bacterium in northeastern China, due to the high infection rate in the tick species and its high abundance. In Hebei province, *A. phagocytophilum* was detected in *H. longicornis* and *D. nuttalli*, where *H. longicornis* is the predominant tick species for the transmission of this pathogen (Yaxue et al., 2011). Phylogenetically, *A. phagocytophilum* detected in the study was more likely to infect ruminants (Yang et al., 2015a).

*Anaplasma bovis* has a wide host range, whose susceptible species include cattle, goats, dogs, cats, and deer (Kocan et al., 2015). In recent years, molecular detection of *A. bovis* infection showed an overall prevalence of 10–16% in goats, and 9.7% in sheep in China (Liu et al., 2012; Zhang et al., unpublished). Both *H. concinna* and *H. longicornis* ticks were found to be infected with *A. bovis*, suggesting these two tick species may be responsible for the transmission of *A. bovis* between ticks and mammals, but this assumption needs further experimental evidence.

*Ehrlichia* spp. are obligate intracellular bacteria residing within the cytoplasmic vacuoles of monocytes, granulocytes, or platelets of humans and animals, and can cause illnesses with fever, leukopenia, and thrombocytopenia (Dumler and Bakken, 1995). Serological and molecular evidences show a wide distribution of the bacteria infection in ticks, animals, and humans (Yu et al., 2016). In China, *E. chaffeensis* was detected in *Amblyomma testudinarium*, *H. yeni*, and *I. persulcatus* (Cao et al., 2000; Zhang et al., 2014). *E. canis* has been identified in *Rhipicephalus sanguineus* and *Rhipicephalus microplus* (Wen et al., 2003), and human infection usually presents fever, malaise, thrombocytopenia, and lymphopenia (Johnson et al., 2015). *E. muris* has been detected in *R. microplus* in Hunan (Yu et al., 2016). In the present study, we first detected *E. muris* in *I. persulcatus* collected from Jilin and Heilongjiang provinces of northeastern China, with a prevalence of 1.9–4.3%, showing that *I. persulcatus* may be a vector for this bacterium in northeastern China. Further studies are needed to assess the possible emergence of the infections in northeastern China.

*Candidatus* N. mikurensis is an emerging tick-borne pathogen causing neoehrlichiosis, whose clinical symptoms may include fever, localized pain in muscles and/or joints, vascular and thromboembolic events (Silaghi et al., 2016). In China, the human infection was first reported in Heilongjiang province of northeastern China in Li et al. (2012), and the bacterium may have wide geographic distribution in China (Li et al., 2013). Only *I. persulcatus* was positive for *Candidatus* N. mikurensis in this study, implying that *I. persulcatus* may be a vector for this bacterium in northeastern China.

Babesiosis, the causative pathogens including *B. microti*, *B. venatorum*, and *B. divergens* in humans, is considered an emerging threat in China, where there are approximately 1.3 billion people at risk of infection (Qi et al., 2011; Jiang et al., 2015; Vannier and Krause, 2015). *B. microti* was detected in *I. persulcatus*, *H. longicornis*, and *H. concinna* in Fujian, Zhejiang, Henan, and Heilongjiang provinces (Saito-Ito et al., 2008; Sun et al., 2008; Zhou et al., 2014). *B. venatorum* has been reported in *I. persulcatus* ticks collected from forested areas of northeastern China (Jiang et al., 2015). Other species of *Babesia*, including *B. ovis*, *B. major*, *B. ovata*, *B. orientalis*, *B. motasi*, and *B. caballi*, have not been shown to infect humans (Fang et al., 2015). In the present study, we found *B. divergens*, *B. microti*, and *B. venatorum* in *I. persulcatus*, and also first detected *B. divergens* in *H. longicornis*, indicating that *I. persulcatus* may be the main vector for the *Babesia* species of human babesiosis in northeastern China, since only two *H. longicornis* pools were positive for this parasite.

Previous studies have shown that *B. microti* is the predominant species in southeastern and northeastern of China while *B. divergens* may be the main pathogen in Inner Mongolia, and Xinjiang Uygur Autonomous Region of China (Zhou et al., 2014). In this study, of the 47 *Babesia*-positive samples, 19 (40.2%) were *B. venatorum*, followed by 9 (19.1%) *B. microti*, 3 (6.4%) *B. divergens*, and 16 (34.3%) sequence variants, suggesting that *B. venatorum* may be the predominant species responsible for human babesiosis in northeastern China.

Additionally, several sequence variants, which are closely related to the groups of carnivores, cattle and small ruminant *Babesia*, have also been found in the present study. For example, *Babesia* sp. Ip-hlj179 was phylogenetically associated with *B. gibsoni*; *Babesia* sp. Ip-hlj179 was related to *B. motasi*; *Babesia* sp. Ip-hlj238 was related to *B. crassa*; and *Babesia* sp. Ip-hlj212 was related to *B. major* (**Figure 3**). Thus, it is necessary to monitor these variants infection in domestic animals in northeastern China. Isolation of parasites and identification of transmission vector should also be included. Interestingly, a high genetic variability of *Babesia* has been described in Russia, which included all variants found in northeastern China (Rar et al., 2014), and four variants were detected in Heilongjiang province while only two variants were found in Jilin province. These data showed the cross-border spread of *Babesia* in northeastern China may occur.

More than 300 *Hepatozoon* species have been identified in amphibians, reptiles, birds, marsupials, and mammals (Smith, 1996). Of these, more than 120 species infect snakes, and approximately 50 have been described in mammals. Ticks and other blood-sucking arthropods may serves as definitive hosts for *Hepatozoon* spp. Unlike, other vector-borne pathogens that are transmitted via the bite of arthropods, the vertebrate host becomes infected by ingestion of the arthropods that contains mature oocysts. Three species *H. canis*, *H. americanum*, and *H. felis* can cause hepatozoonosis in dogs and cats, showing different clinical symptoms. *H. canis* is primarily found in hemolymphatic tissues, causing fever, lethargy, weight loss, anemia, and hyperglobulinemia in dogs, while *H. americanum* infects mainly muscular tissues, causing myositis and lameness (Baneth, 2011). There is only one report of infection with a *Hepatozoon* sp. in a person from Russia; the patient was anemic and icteric, and gamonts were detected in the blood (Shuikina et al., 2004).

In China, *H. canis* infection in dogs was detected 1.1% in Jiangsu, 1.2% in Xinjiang, 8.9% in Shaanxi, and 4.9% in Henan and Beijing (Xu et al., 2015). A new species, *H. chinensis* has been found in king rat snakes (*Elaphe carinata*) from Shanghai (Han et al., 2015). In this study, two *Hepatozoon* species, the most closely related to the isolates of Japanese martens, were found in ticks in northeastern China, and both of them were detected in *Ixodes*, *Haemaphysalis*, and *Dermacentor* ticks, suggesting that specificity for the final host may be low in *Hepatozoon*; however, the intermediate hosts and the resulting disease still remain to be determined.

Not only the high infection rates but also the high abundance of *I. persulcatus* makes it the most important vector tick in the area. Moreover, *Candidatus* N. mikurensis, *B. venatorum*, and *B. microti* were only detected in *I. persulcatus.* These findings show that *I. persulcatus* may be an important vector of tick-borne bacteria and protozoa in northeastern China.

In summary, we detected four species of bacteria and three species of protozoa in four tick species in northeastern China, including *A. phagocytophilum*, *A. bovis*, *E. muris*, *Candidatus* N. mikurensis, *B. venatorum*, *B. microti*, and *B. divergens*, which are associated with emerging diseases in humans and/or animals. Additionally, four *Babesia* sequence variants, and two *Hepatozoon* sp. were also found. These findings showed the genetic variability of *Anaplasma*, *Ehrlichia*, *Babesia*, and *Hepatozoon* spp. circulating in ticks in northeastern China, highlighting the need for further research of these tick-associated pathogens and their role in human and animal diseases. Further studies will be necessary to confirm the vectorial capacity of ticks, to improve understanding of the epidemiology of these tick-borne diseases, and to monitor emerging tick-borne pathogens and factors influencing their prevalence, which will facilitate implementing integrated strategies for controlling ticks and tick-borne pathogens in China.

## Author Contributions

JQ and QL designed the study in collaboration with FW, MS, and HL. HM conducted the fieldwork with assistance from MS, ZZ, and QL. HL, BW, ZW, and ZL conducted the laboratory work; HL and SW conducted the statistical analysis and drafted the manuscript. FW and MS contributed to the interpretation of the data. All authors contributed to the manuscript editing and approved the final manuscript.

## Conflict of Interest Statement

The authors declare that the research was conducted in the absence of any commercial or financial relationships that could be construed as a potential conflict of interest.

## Footnotes

1 http://www.megasoftware.net/

## Supplementary Material

FIGURE S1 **Sampling sites of ticks collected in Jilin and Heilongjiang provinces of northeastern China.** Tick species and the number are shown.
