# Supplementary figures and images for: Molecular Detection and Characterization of Zoonotic and Veterinary Pathogens in Ticks from Northeastern China
Source: Front Microbiol. 2016 Nov 29;7:1913. doi: 10.3389/fmicb.2016.01913 (PMC5126052; doi:10.3389/fmicb.2016.01913)

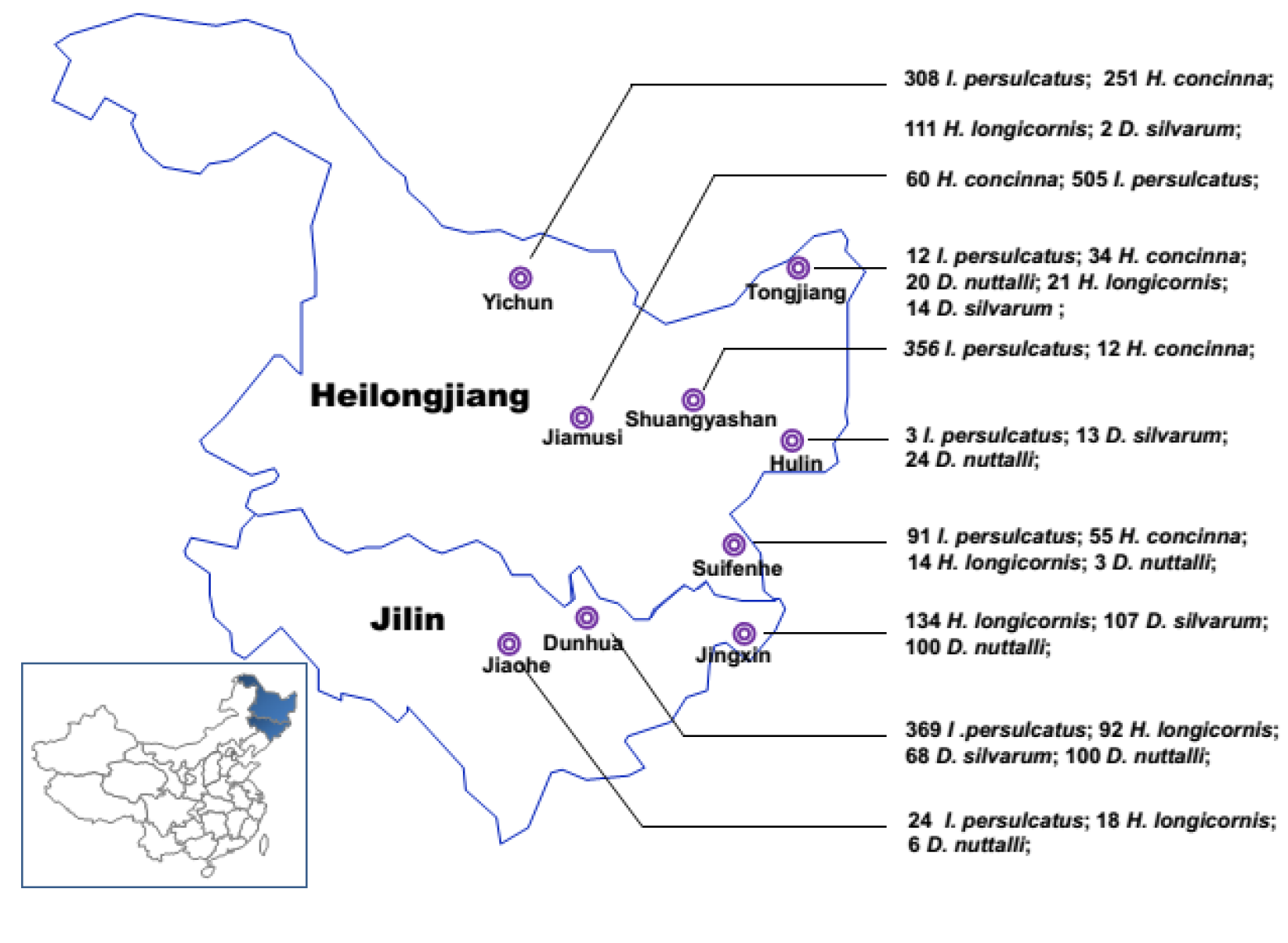

Supplement: FIGURE S1 — Sampling sites of ticks collected in Jilin and Heilongjiang provinces of northeastern China. Tick species and the number are shown. [file Image_1.TIF]
