# Supplementary material for: Molecular Detection and Characterization of Zoonotic and Veterinary Pathogens in Ticks from Northeastern China
Source: Front Microbiol. 2016 Nov 29;7:1913. doi: 10.3389/fmicb.2016.01913 (PMC5126052; doi:10.3389/fmicb.2016.01913)
Supplement: Supplementary file 2 [file Table_1.DOCX]

**Supplementary Table 1.** The evolutionary divergence between sequences of *Anaplasma*.

|  | | 1 | 2 | 3 | 4 | 5 | 6 | 7 | 8 | 9 | 10 | 11 | 12 | 13 | 14 | 15 | 16 | 17 | 18 | 19 | 20 |
| --- | --- | --- | --- | --- | --- | --- | --- | --- | --- | --- | --- | --- | --- | --- | --- | --- | --- | --- | --- | --- | --- |
| 1 | **A. bovis,KU921422** |  |  |  |  |  |  |  |  |  |  |  |  |  |  |  |  |  |  |  |  |
| 2 | **A. phagocytophilum,KX810088** | 0.02 |  |  |  |  |  |  |  |  |  |  |  |  |  |  |  |  |  |  |  |
| 3 | A. bovis, LC012812 | 0.00 | 0.02 |  |  |  |  |  |  |  |  |  |  |  |  |  |  |  |  |  |  |
| 4 | A. bovis, JX092092 | 0.00 | 0.02 | 0.00 |  |  |  |  |  |  |  |  |  |  |  |  |  |  |  |  |  |
| 5 | A. bovis, KM114612 | 0.00 | 0.02 | 0.00 | 0.00 |  |  |  |  |  |  |  |  |  |  |  |  |  |  |  |  |
| 6 | A. bovis, KP062958 | 0.00 | 0.02 | 0.00 | 0.00 | 0.00 |  |  |  |  |  |  |  |  |  |  |  |  |  |  |  |
| 7 | A. bovis, FJ169957 | 0.00 | 0.02 | 0.00 | 0.00 | 0.00 | 0.00 |  |  |  |  |  |  |  |  |  |  |  |  |  |  |
| 8 | A. bovis, KC335228 | 0.00 | 0.02 | 0.00 | 0.00 | 0.00 | 0.00 | 0.00 |  |  |  |  |  |  |  |  |  |  |  |  |  |
| 9 | A. centrale, KU686784 | 0.03 | 0.02 | 0.03 | 0.03 | 0.03 | 0.03 | 0.03 | 0.03 |  |  |  |  |  |  |  |  |  |  |  |  |
| 10 | A. marginale, KU686794 | 0.03 | 0.02 | 0.03 | 0.03 | 0.03 | 0.03 | 0.03 | 0.03 | 0.00 |  |  |  |  |  |  |  |  |  |  |  |
| 11 | A. ovis, AY262124 | 0.03 | 0.02 | 0.03 | 0.03 | 0.03 | 0.03 | 0.03 | 0.03 | 0.00 | 0.00 |  |  |  |  |  |  |  |  |  |  |
| 12 | A. phagocytophilum, KF569911 | 0.02 | 0.00 | 0.02 | 0.02 | 0.02 | 0.02 | 0.02 | 0.02 | 0.02 | 0.02 | 0.02 |  |  |  |  |  |  |  |  |  |
| 13 | A. phagocytophilum, HM366589 | 0.02 | 0.01 | 0.02 | 0.02 | 0.02 | 0.01 | 0.01 | 0.01 | 0.02 | 0.02 | 0.02 | 0.01 |  |  |  |  |  |  |  |  |
| 14 | A. phagocytophilum, DQ458808 | 0.02 | 0.01 | 0.02 | 0.02 | 0.02 | 0.01 | 0.01 | 0.01 | 0.02 | 0.02 | 0.02 | 0.01 | 0.00 |  |  |  |  |  |  |  |
| 15 | A. phagocytophilum, AB196721 | 0.02 | 0.00 | 0.02 | 0.02 | 0.02 | 0.02 | 0.02 | 0.02 | 0.03 | 0.03 | 0.03 | 0.00 | 0.01 | 0.01 |  |  |  |  |  |  |
| 16 | A. phagocytophilum, KP062963 | 0.02 | 0.00 | 0.02 | 0.02 | 0.02 | 0.02 | 0.02 | 0.02 | 0.02 | 0.02 | 0.02 | 0.00 | 0.01 | 0.01 | 0.00 |  |  |  |  |  |
| 17 | A. phagocytophilum, KP306518 | 0.02 | 0.01 | 0.02 | 0.02 | 0.02 | 0.01 | 0.01 | 0.01 | 0.02 | 0.02 | 0.02 | 0.01 | 0.00 | 0.00 | 0.01 | 0.01 |  |  |  |  |
| 18 | A. phagocytophilum, AY776165 | 0.02 | 0.01 | 0.02 | 0.02 | 0.02 | 0.01 | 0.01 | 0.01 | 0.02 | 0.02 | 0.02 | 0.01 | 0.00 | 0.00 | 0.01 | 0.01 | 0.00 |  |  |  |
| 19 | Anaplasma sp.sh65-5, KM186934 | 0.00 | 0.02 | 0.00 | 0.00 | 0.00 | 0.00 | 0.00 | 0.00 | 0.03 | 0.03 | 0.03 | 0.02 | 0.02 | 0.02 | 0.02 | 0.02 | 0.02 | 0.02 |  |  |
| 20 | A. phagocytophilum, U02521 | 0.02 | 0.01 | 0.02 | 0.02 | 0.02 | 0.01 | 0.01 | 0.01 | 0.02 | 0.02 | 0.02 | 0.01 | 0.00 | 0.00 | 0.01 | 0.01 | 0.00 | 0.00 | 0.02 |  |
| 21 | A. phagocytophilum, DQ342324 | 0.02 | 0.01 | 0.02 | 0.02 | 0.02 | 0.01 | 0.01 | 0.01 | 0.02 | 0.02 | 0.02 | 0.01 | 0.00 | 0.00 | 0.01 | 0.01 | 0.00 | 0.00 | 0.02 | 0.00 |
