# Supplementary material for: Molecular Detection and Characterization of Zoonotic and Veterinary Pathogens in Ticks from Northeastern China
Source: Front Microbiol. 2016 Nov 29;7:1913. doi: 10.3389/fmicb.2016.01913 (PMC5126052; doi:10.3389/fmicb.2016.01913)
Supplement: Supplementary file 3 [file Table_2.DOCX]

**Supplementary Table 2.** Detection of *Anaplasma* spp. in ticks from northeastern China by nested PCR.

| Tick species | No. of ticks tested (%) | Prevalence (%, 95% CI) | Jilin | | | Heilongjiang^*^ | | | | |
| --- | --- | --- | --- | --- | --- | --- | --- | --- | --- | --- |
|  |  |  | Subtotal no. ticks tested | no. positive pools/ No. pool | *A.p* (%, 95% CI) |  | Subtotal no. ticks tested | no. positive pools/no. pools | *A.p* (%, 95% CI) | *A.b* (%, 95% CI) |
| *D. nuttalli* | 253 (8.0) | 1.6 (0.5-3.8)^a^ | 206 | 1/16 | 0.5 (0.1-2.3) |  | 47 | 3/5 | 7.1 (2.2-20.0) | 0 |
| *D. silvarum* | 204 (7.0) | 2.3 (0.8-5.7) ^b^ | 175 | 2/11 | 2.2 (0.8-5.3) |  | 29 | 2/3 | 6.1 (1.7-24.4) | 0 |
| *H. concinna* | 412 (14.0) | 7.2 (4.5-11.3) | 0 | 0 | 0 |  | 412 | 19/28 | 6.5 (4.1-10.3) | 0.2 (0.1-1.2) |
| *H. longicornis* | 390 (13.0) | 3.6 (2.0-6.2)^c^ | 244 | 7/15 | 3.7 (1.8-7.3) |  | 146 | 4/11 | 2.3 (0.6-6.1) | 0.7 (0.1-3.3) |
| *I. persulcatus* | 1669 (58.0) | 9.1 (7.4-11.3)^a,b,c^ | 393 | 24/27 | 12.6 (8.3-20.1) |  | 1276 | 63/88 | 8.2 (6.4-10.6) | 0 |
| Total | 2928 | 6.4 (5.4-7.6) | 1018 | 34/69 | 4.5 (3.2-6.2)^d^ |  | 1910 | 91/135 | 7.2 (5.9-9.0)^d^ | 0.1 (0.0-0.3) |

^*^*A.p*: *Anaplasma phagocytophilum*; *A.b*: *Anaplasma bovis*;

^a,b,c,d^ Significant difference was found between the prevalence in the two groups (p<0.05), analyzed by the Fisher's exact test.
