# Supplementary material for: Molecular Detection and Characterization of Zoonotic and Veterinary Pathogens in Ticks from Northeastern China
Source: Front Microbiol. 2016 Nov 29;7:1913. doi: 10.3389/fmicb.2016.01913 (PMC5126052; doi:10.3389/fmicb.2016.01913)
Supplement: Supplementary file 4 [file Table_3.DOCX]

**Supplementary Table 3.** The evolutionary divergence among the sequences of *Ehrlichia*.

|  | | 1 | 2 | 3 | 4 | 5 | 6 | 7 | 8 | 9 | 10 | 11 | 12 | 13 | 14 | 15 | 16 | 17 | 18 | 19 | 20 | 21 | 22 | 23 | 24 |
| --- | --- | --- | --- | --- | --- | --- | --- | --- | --- | --- | --- | --- | --- | --- | --- | --- | --- | --- | --- | --- | --- | --- | --- | --- | --- |
| 1 | **Ehrlichia sp. hc-hlj209, KU921424** |  |  |  |  |  |  |  |  |  |  |  |  |  |  |  |  |  |  |  |  |  |  |  |  |
| 2 | **Candidatus N. mikurensis, KU921420** | 0.26 |  |  |  |  |  |  |  |  |  |  |  |  |  |  |  |  |  |  |  |  |  |  |  |
| 3 | **E. muris, KU921423** | 0.09 | 0.24 |  |  |  |  |  |  |  |  |  |  |  |  |  |  |  |  |  |  |  |  |  |  |
| 4 | Ehrlichia sp. P-Mtn, HQ658904 | 0.11 | 0.24 | 0.14 |  |  |  |  |  |  |  |  |  |  |  |  |  |  |  |  |  |  |  |  |  |
| 5 | Candidatus E. khabarensis, KR063139 | 0.11 | 0.22 | 0.11 | 0.15 |  |  |  |  |  |  |  |  |  |  |  |  |  |  |  |  |  |  |  |  |
| 6 | Candidatus E. ovata, DQ672553 | 0.09 | 0.28 | 0.04 | 0.14 | 0.11 |  |  |  |  |  |  |  |  |  |  |  |  |  |  |  |  |  |  |  |
| 7 | Candidatus E. regneryi, KJ814961 | 0.11 | 0.29 | 0.08 | 0.13 | 0.14 | 0.09 |  |  |  |  |  |  |  |  |  |  |  |  |  |  |  |  |  |  |
| 8 | Ehrlichia sp. Am-Hc79, JX092091 | 0.03 | 0.29 | 0.09 | 0.12 | 0.13 | 0.10 | 0.11 |  |  |  |  |  |  |  |  |  |  |  |  |  |  |  |  |  |
| 9 | Ehrlichia sp. Kh-Hj27, FJ966349 | 0.00 | 0.26 | 0.09 | 0.11 | 0.11 | 0.09 | 0.11 | 0.03 |  |  |  |  |  |  |  |  |  |  |  |  |  |  |  |  |
| 10 | Candidatus E. shimanensis, AB074462 | 0.09 | 0.22 | 0.10 | 0.12 | 0.11 | 0.08 | 0.10 | 0.09 | 0.09 |  |  |  |  |  |  |  |  |  |  |  |  |  |  |  |
| 11 | E. canis, JN391408 | 0.12 | 0.28 | 0.10 | 0.14 | 0.14 | 0.10 | 0.04 | 0.13 | 0.12 | 0.10 |  |  |  |  |  |  |  |  |  |  |  |  |  |  |
| 12 | Candidatus N. mikurensis, FJ966359 | 0.26 | 0.00 | 0.24 | 0.24 | 0.22 | 0.28 | 0.29 | 0.29 | 0.26 | 0.22 | 0.28 |  |  |  |  |  |  |  |  |  |  |  |  |  |
| 13 | Candidatus N. mikurensis, EU432375 | 0.26 | 0.01 | 0.24 | 0.24 | 0.23 | 0.28 | 0.28 | 0.29 | 0.26 | 0.23 | 0.29 | 0.01 |  |  |  |  |  |  |  |  |  |  |  |  |
| 14 | Candidatus N. mikurensis, KF803997 | 0.26 | 0.01 | 0.24 | 0.24 | 0.23 | 0.28 | 0.28 | 0.29 | 0.26 | 0.23 | 0.29 | 0.01 | 0.00 |  |  |  |  |  |  |  |  |  |  |  |
| 15 | Candidatus N. mikurensis, LC167302 | 0.26 | 0.01 | 0.24 | 0.24 | 0.23 | 0.28 | 0.28 | 0.29 | 0.26 | 0.23 | 0.29 | 0.01 | 0.00 | 0.00 |  |  |  |  |  |  |  |  |  |  |
| 16 | Candidatus N. mikurensis, KJ663733 | 0.26 | 0.01 | 0.24 | 0.24 | 0.23 | 0.28 | 0.28 | 0.29 | 0.26 | 0.23 | 0.29 | 0.01 | 0.00 | 0.00 | 0.00 |  |  |  |  |  |  |  |  |  |
| 17 | Candidatus N. mikurensis, AB204864 | 0.26 | 0.00 | 0.24 | 0.24 | 0.22 | 0.28 | 0.29 | 0.29 | 0.26 | 0.22 | 0.28 | 0.00 | 0.01 | 0.01 | 0.01 | 0.01 |  |  |  |  |  |  |  |  |
| 18 | Candidatus N. lotoris, EF633745 | 0.24 | 0.08 | 0.24 | 0.24 | 0.21 | 0.27 | 0.24 | 0.25 | 0.24 | 0.23 | 0.25 | 0.08 | 0.09 | 0.09 | 0.09 | 0.09 | 0.08 |  |  |  |  |  |  |  |
| 19 | Candidatus N. mikurensis, JQ359062 | 0.26 | 0.00 | 0.24 | 0.24 | 0.22 | 0.28 | 0.29 | 0.29 | 0.26 | 0.22 | 0.28 | 0.00 | 0.01 | 0.01 | 0.01 | 0.01 | 0.00 | 0.08 |  |  |  |  |  |  |
| 20 | E. ewingii, AF195273 | 0.04 | 0.26 | 0.09 | 0.10 | 0.12 | 0.10 | 0.10 | 0.05 | 0.04 | 0.09 | 0.12 | 0.26 | 0.26 | 0.26 | 0.26 | 0.26 | 0.26 | 0.23 | 0.26 |  |  |  |  |  |
| 21 | E. chaffeensis, CP007480 | 0.08 | 0.25 | 0.08 | 0.12 | 0.10 | 0.08 | 0.09 | 0.09 | 0.08 | 0.09 | 0.09 | 0.25 | 0.25 | 0.25 | 0.25 | 0.25 | 0.25 | 0.21 | 0.25 | 0.09 |  |  |  |  |
| 22 | E. ruminantium, AB625794 | 0.16 | 0.29 | 0.19 | 0.13 | 0.19 | 0.19 | 0.18 | 0.15 | 0.16 | 0.17 | 0.20 | 0.29 | 0.29 | 0.29 | 0.29 | 0.29 | 0.29 | 0.26 | 0.29 | 0.15 | 0.16 |  |  |  |
| 23 | E. muris, KF312362 | 0.09 | 0.24 | 0.00 | 0.14 | 0.11 | 0.04 | 0.08 | 0.09 | 0.09 | 0.10 | 0.10 | 0.24 | 0.24 | 0.24 | 0.24 | 0.24 | 0.24 | 0.24 | 0.24 | 0.09 | 0.08 | 0.19 |  |  |
| 24 | E. muris, AB204863 | 0.09 | 0.24 | 0.00 | 0.14 | 0.11 | 0.04 | 0.08 | 0.09 | 0.09 | 0.10 | 0.10 | 0.24 | 0.24 | 0.24 | 0.24 | 0.24 | 0.24 | 0.24 | 0.24 | 0.09 | 0.08 | 0.19 | 0.00 |  |
| 25 | E. muris, GU358690 | 0.09 | 0.24 | 0.00 | 0.14 | 0.11 | 0.04 | 0.08 | 0.09 | 0.09 | 0.10 | 0.10 | 0.24 | 0.24 | 0.24 | 0.24 | 0.24 | 0.24 | 0.24 | 0.24 | 0.09 | 0.08 | 0.19 | 0.00 | 0.00 |
