# Supplementary material for: Molecular Detection and Characterization of Zoonotic and Veterinary Pathogens in Ticks from Northeastern China
Source: Front Microbiol. 2016 Nov 29;7:1913. doi: 10.3389/fmicb.2016.01913 (PMC5126052; doi:10.3389/fmicb.2016.01913)
Supplement: Supplementary file 5 [file Table_4.DOCX]

**Supplementary Table 4.** Detection of *Ehrlichia* spp. in ticks from northeastern China by nested PCR.

| Tick species | No. of ticks tested | Prevalence  (%, 95% CI) | Heilongjiang* | | | | |  | Jilin | | | |
| --- | --- | --- | --- | --- | --- | --- | --- | --- | --- | --- | --- | --- |
|  |  |  | Subtotal no. ticks tested | No. positive pools/ no. pools | E.m (%, 95% CI ) | C.N.m (%, 95% CI) | E.hc-hlj209 (%, 95% CI ) |  | Subtotal no. ticks tested | No. positive pools/ no. pools | E.m (%, 95% CI ) | C.N.m (%, 95% CI ) |
| *D. nuttalli* | 253 | 0 | 47 | 0/5 | 0 | 0 | 0 |  | 206 | 0/16 | 0 | 0 |
| *D. silvarum* | 204 | 0 | 29 | 0/3 | 0 | 0 | 0 |  | 175 | 0/11 | 0 | 0 |
| *H. concinna* | 412 | 0.5 (0.1-1.6)^a^ | 412 | 2/28 | 0.2 (0.1-1.2) | 0 | 0.2 (0.1-1.2) |  | 0 | 0 | 0 | 0 |
| *H. longicornis* | 390 | 0.8 (0.2-2.1) | 146 | 3/11 | 0 | 0 | 2.2 (0.6-6.1) |  | 244 | 0/15 | 0 | 0 |
| *I .persulcatus* | 1669 | 3.1 (2.2-4.1)^a^ | 1276 | 28/88 | 1.9 (1.3-2.9)^b^ | 0.5 (0.1-1.7) | 0 |  | 393 | 14/27 | 4.3 (2.4-7.2)^b^ | 0.3 (0.1-1.2) |
| Total | 2928 | 1.8 (1.3-2.4) | 1910 | 33/135 | 1.3 (0.9-1.9) | 0.3 (0.1-0.7) | 0.2 (0.1-0.5) |  | 1018 | 14/69 | 1.4 (0.8-2.3) | 0.1 (0.0-0.5) |

*E.m, Ehrlichia muris (KU921423); C.N.m, Candidatus Neoehrlichia mikurensis (KU921420); E.hc-hlj209: *Ehrlichia* sp.hc-hlj209 (KU921424) detected in *H. concinna* collected in Heilongjiang.

^a,b^Significant difference was found between the prevalence in the two tick species (p<0.05), analyzed by the Fisher's exact test.
