# Supplementary material for: Molecular Detection and Characterization of Zoonotic and Veterinary Pathogens in Ticks from Northeastern China
Source: Front Microbiol. 2016 Nov 29;7:1913. doi: 10.3389/fmicb.2016.01913 (PMC5126052; doi:10.3389/fmicb.2016.01913)
Supplement: Supplementary file 6 [file Table_5.DOCX]

**Supplementary Table 5.** The evolutionary divergence between sequences of *Babesia*.

|  |  | 1 | 2 | 3 | 4 | 5 | 6 | 7 | 8 | 9 | 10 | 11 | 12 | 13 | 14 | 15 | 16 | 17 | 18 | 19 | 20 | 21 | 22 | 23 | 24 | 25 | 26 | 27 | 28 | 29 | 30 | 31 | 32 | 33 | 34 | 35 | 36 | 37 |
| --- | --- | --- | --- | --- | --- | --- | --- | --- | --- | --- | --- | --- | --- | --- | --- | --- | --- | --- | --- | --- | --- | --- | --- | --- | --- | --- | --- | --- | --- | --- | --- | --- | --- | --- | --- | --- | --- | --- |
| 1 | **B. divergens, KU862300** |  |  |  |  |  |  |  |  |  |  |  |  |  |  |  |  |  |  |  |  |  |  |  |  |  |  |  |  |  |  |  |  |  |  |  |  |  |
| 2 | **B. microti, KU862301** | 0.08 |  |  |  |  |  |  |  |  |  |  |  |  |  |  |  |  |  |  |  |  |  |  |  |  |  |  |  |  |  |  |  |  |  |  |  |  |
| 3 | **B. venatorum, KU862302** | 0.01 | 0.08 |  |  |  |  |  |  |  |  |  |  |  |  |  |  |  |  |  |  |  |  |  |  |  |  |  |  |  |  |  |  |  |  |  |  |  |
| 4 | **Babesia sp. Ip-hlj179, KU862305** | 0.03 | 0.09 | 0.02 |  |  |  |  |  |  |  |  |  |  |  |  |  |  |  |  |  |  |  |  |  |  |  |  |  |  |  |  |  |  |  |  |  |  |
| 5 | **Babesia sp. Ip-hlj238, KU862303** | 0.05 | 0.08 | 0.05 | 0.06 |  |  |  |  |  |  |  |  |  |  |  |  |  |  |  |  |  |  |  |  |  |  |  |  |  |  |  |  |  |  |  |  |  |
| 6 | **Babesia sp. hl-hlj178, KU862306** | 0.06 | 0.09 | 0.06 | 0.06 | 0.04 |  |  |  |  |  |  |  |  |  |  |  |  |  |  |  |  |  |  |  |  |  |  |  |  |  |  |  |  |  |  |  |  |
| 7 | **Babeisia sp. hc-hlj212, KU862304** | 0.05 | 0.08 | 0.05 | 0.05 | 0.02 | 0.03 |  |  |  |  |  |  |  |  |  |  |  |  |  |  |  |  |  |  |  |  |  |  |  |  |  |  |  |  |  |  |  |
| 8 | B.bigemina, FJ426361 | 0.05 | 0.08 | 0.04 | 0.04 | 0.03 | 0.04 | 0.02 |  |  |  |  |  |  |  |  |  |  |  |  |  |  |  |  |  |  |  |  |  |  |  |  |  |  |  |  |  |  |
| 9 | B. canis, AB083374 | 0.03 | 0.09 | 0.02 | 0.03 | 0.06 | 0.07 | 0.06 | 0.06 |  |  |  |  |  |  |  |  |  |  |  |  |  |  |  |  |  |  |  |  |  |  |  |  |  |  |  |  |  |
| 10 | B. capreoli, AY726009 | 0.00 | 0.08 | 0.01 | 0.03 | 0.05 | 0.06 | 0.05 | 0.05 | 0.03 |  |  |  |  |  |  |  |  |  |  |  |  |  |  |  |  |  |  |  |  |  |  |  |  |  |  |  |  |
| 11 | B. crassa, AY260177 | 0.04 | 0.08 | 0.05 | 0.05 | 0.02 | 0.04 | 0.02 | 0.03 | 0.06 | 0.04 |  |  |  |  |  |  |  |  |  |  |  |  |  |  |  |  |  |  |  |  |  |  |  |  |  |  |  |
| 12 | B. crassa, AY260176 | 0.05 | 0.09 | 0.05 | 0.05 | 0.02 | 0.04 | 0.02 | 0.04 | 0.06 | 0.05 | 0.02 |  |  |  |  |  |  |  |  |  |  |  |  |  |  |  |  |  |  |  |  |  |  |  |  |  |  |
| 13 | B. crassa, JX542614 | 0.05 | 0.08 | 0.05 | 0.05 | 0.01 | 0.03 | 0.01 | 0.03 | 0.06 | 0.05 | 0.02 | 0.01 |  |  |  |  |  |  |  |  |  |  |  |  |  |  |  |  |  |  |  |  |  |  |  |  |  |
| 14 | B. divergens, FJ944826 | 0.00 | 0.08 | 0.01 | 0.03 | 0.05 | 0.06 | 0.05 | 0.05 | 0.03 | 0.00 | 0.04 | 0.05 | 0.05 |  |  |  |  |  |  |  |  |  |  |  |  |  |  |  |  |  |  |  |  |  |  |  |  |
| 15 | B. divergens, GU057385 | 0.00 | 0.08 | 0.01 | 0.03 | 0.05 | 0.06 | 0.05 | 0.05 | 0.03 | 0.00 | 0.04 | 0.05 | 0.05 | 0.00 |  |  |  |  |  |  |  |  |  |  |  |  |  |  |  |  |  |  |  |  |  |  |  |
| 16 | B. divergens, KJ486559 | 0.00 | 0.08 | 0.01 | 0.03 | 0.05 | 0.06 | 0.05 | 0.05 | 0.03 | 0.00 | 0.04 | 0.05 | 0.05 | 0.00 | 0.00 |  |  |  |  |  |  |  |  |  |  |  |  |  |  |  |  |  |  |  |  |  |  |
| 17 | B. gibsoni, AB118032 | 0.03 | 0.09 | 0.02 | 0.02 | 0.05 | 0.06 | 0.05 | 0.05 | 0.03 | 0.03 | 0.04 | 0.05 | 0.05 | 0.03 | 0.03 | 0.03 |  |  |  |  |  |  |  |  |  |  |  |  |  |  |  |  |  |  |  |  |  |
| 18 | B. gibsoni, FJ769386 | 0.03 | 0.09 | 0.02 | 0.02 | 0.05 | 0.06 | 0.05 | 0.05 | 0.03 | 0.03 | 0.04 | 0.05 | 0.05 | 0.03 | 0.03 | 0.03 | 0.00 |  |  |  |  |  |  |  |  |  |  |  |  |  |  |  |  |  |  |  |  |
| 19 | B. major, EU622907 | 0.06 | 0.09 | 0.05 | 0.05 | 0.03 | 0.05 | 0.02 | 0.04 | 0.06 | 0.06 | 0.03 | 0.03 | 0.02 | 0.06 | 0.06 | 0.06 | 0.05 | 0.05 |  |  |  |  |  |  |  |  |  |  |  |  |  |  |  |  |  |  |  |
| 20 | B. microti, AB032434 | 0.08 | 0.01 | 0.08 | 0.08 | 0.08 | 0.08 | 0.08 | 0.08 | 0.09 | 0.08 | 0.08 | 0.08 | 0.08 | 0.08 | 0.08 | 0.08 | 0.08 | 0.08 | 0.09 |  |  |  |  |  |  |  |  |  |  |  |  |  |  |  |  |  |  |
| 21 | B. microti, AF231348 | 0.08 | 0.00 | 0.08 | 0.09 | 0.08 | 0.09 | 0.08 | 0.08 | 0.09 | 0.08 | 0.08 | 0.09 | 0.08 | 0.08 | 0.08 | 0.08 | 0.09 | 0.09 | 0.09 | 0.01 |  |  |  |  |  |  |  |  |  |  |  |  |  |  |  |  |  |
| 22 | B. microti, LC005772 | 0.08 | 0.00 | 0.08 | 0.09 | 0.08 | 0.09 | 0.08 | 0.08 | 0.09 | 0.08 | 0.08 | 0.09 | 0.08 | 0.08 | 0.08 | 0.08 | 0.09 | 0.09 | 0.09 | 0.01 | 0.00 |  |  |  |  |  |  |  |  |  |  |  |  |  |  |  |  |
| 23 | B. microti, AY943957 | 0.08 | 0.00 | 0.08 | 0.09 | 0.08 | 0.09 | 0.08 | 0.08 | 0.09 | 0.08 | 0.08 | 0.09 | 0.08 | 0.08 | 0.08 | 0.08 | 0.09 | 0.09 | 0.09 | 0.01 | 0.00 | 0.00 |  |  |  |  |  |  |  |  |  |  |  |  |  |  |  |
| 24 | Babesia_motasi_AY260179 | 0.06 | 0.09 | 0.06 | 0.06 | 0.04 | 0.01 | 0.03 | 0.04 | 0.07 | 0.06 | 0.04 | 0.04 | 0.04 | 0.06 | 0.06 | 0.06 | 0.06 | 0.06 | 0.04 | 0.08 | 0.09 | 0.09 | 0.09 |  |  |  |  |  |  |  |  |  |  |  |  |  |  |
| 25 | B. odocoilei, KC460321 | 0.01 | 0.08 | 0.01 | 0.02 | 0.05 | 0.06 | 0.05 | 0.04 | 0.03 | 0.01 | 0.04 | 0.05 | 0.05 | 0.01 | 0.01 | 0.01 | 0.02 | 0.02 | 0.05 | 0.08 | 0.08 | 0.08 | 0.08 | 0.06 |  |  |  |  |  |  |  |  |  |  |  |  |  |
| 26 | B. ovata, AY603400 | 0.05 | 0.08 | 0.04 | 0.04 | 0.03 | 0.04 | 0.02 | 0.01 | 0.05 | 0.05 | 0.03 | 0.03 | 0.03 | 0.05 | 0.05 | 0.05 | 0.04 | 0.04 | 0.04 | 0.08 | 0.08 | 0.08 | 0.08 | 0.04 | 0.04 |  |  |  |  |  |  |  |  |  |  |  |  |
| 27 | Babesia sp. Irk-Hc130, KJ486569 | 0.06 | 0.09 | 0.06 | 0.06 | 0.04 | 0.00 | 0.03 | 0.04 | 0.07 | 0.06 | 0.04 | 0.04 | 0.03 | 0.06 | 0.06 | 0.06 | 0.06 | 0.06 | 0.05 | 0.08 | 0.09 | 0.09 | 0.09 | 0.01 | 0.06 | 0.04 |  |  |  |  |  |  |  |  |  |  |  |
| 28 | Babesia sp. Irk-Ip525, KJ486566 | 0.05 | 0.08 | 0.05 | 0.06 | 0.00 | 0.04 | 0.02 | 0.03 | 0.06 | 0.05 | 0.02 | 0.02 | 0.01 | 0.05 | 0.05 | 0.05 | 0.05 | 0.05 | 0.03 | 0.08 | 0.08 | 0.08 | 0.08 | 0.04 | 0.05 | 0.03 | 0.04 |  |  |  |  |  |  |  |  |  |  |
| 29 | Babesia sp. Kh-Hc222, KJ486568 | 0.05 | 0.08 | 0.05 | 0.05 | 0.02 | 0.03 | 0.00 | 0.02 | 0.06 | 0.05 | 0.02 | 0.02 | 0.01 | 0.05 | 0.05 | 0.05 | 0.05 | 0.05 | 0.02 | 0.08 | 0.08 | 0.08 | 0.08 | 0.03 | 0.05 | 0.02 | 0.03 | 0.02 |  |  |  |  |  |  |  |  |  |
| 30 | Babesia sp. Kh-Hj131, GU057381 | 0.05 | 0.08 | 0.05 | 0.05 | 0.01 | 0.03 | 0.01 | 0.03 | 0.06 | 0.05 | 0.02 | 0.01 | 0.00 | 0.05 | 0.05 | 0.05 | 0.05 | 0.05 | 0.02 | 0.08 | 0.08 | 0.08 | 0.08 | 0.04 | 0.05 | 0.03 | 0.03 | 0.01 | 0.01 |  |  |  |  |  |  |  |  |
| 31 | Babesia sp. Kh-Hj143, GU057382 | 0.03 | 0.09 | 0.02 | 0.00 | 0.06 | 0.06 | 0.05 | 0.04 | 0.03 | 0.03 | 0.05 | 0.05 | 0.05 | 0.03 | 0.03 | 0.03 | 0.02 | 0.02 | 0.05 | 0.08 | 0.09 | 0.09 | 0.09 | 0.06 | 0.02 | 0.04 | 0.06 | 0.06 | 0.05 | 0.05 |  |  |  |  |  |  |  |
| 32 | Babesia sp. Kh-Hj441, KJ486570 | 0.02 | 0.08 | 0.02 | 0.01 | 0.05 | 0.06 | 0.05 | 0.04 | 0.03 | 0.02 | 0.04 | 0.05 | 0.05 | 0.02 | 0.02 | 0.02 | 0.02 | 0.02 | 0.05 | 0.07 | 0.08 | 0.08 | 0.08 | 0.06 | 0.02 | 0.04 | 0.06 | 0.05 | 0.05 | 0.05 | 0.01 |  |  |  |  |  |  |
| 33 | Babesia sp. MO1, AY048113 | 0.00 | 0.09 | 0.01 | 0.03 | 0.06 | 0.06 | 0.05 | 0.05 | 0.03 | 0.00 | 0.05 | 0.05 | 0.06 | 0.00 | 0.00 | 0.00 | 0.03 | 0.03 | 0.06 | 0.08 | 0.09 | 0.09 | 0.09 | 0.07 | 0.01 | 0.05 | 0.06 | 0.06 | 0.05 | 0.06 | 0.03 | 0.02 |  |  |  |  |  |
| 34 | B.venatorum, FJ215873 | 0.01 | 0.08 | 0.00 | 0.02 | 0.05 | 0.06 | 0.05 | 0.04 | 0.02 | 0.01 | 0.05 | 0.05 | 0.05 | 0.01 | 0.01 | 0.01 | 0.02 | 0.02 | 0.05 | 0.08 | 0.08 | 0.08 | 0.08 | 0.06 | 0.01 | 0.04 | 0.06 | 0.05 | 0.05 | 0.05 | 0.02 | 0.02 | 0.01 |  |  |  |  |
| 35 | B. venatorum, KJ486557 | 0.01 | 0.08 | 0.00 | 0.02 | 0.05 | 0.06 | 0.05 | 0.04 | 0.02 | 0.01 | 0.05 | 0.05 | 0.05 | 0.01 | 0.01 | 0.01 | 0.02 | 0.02 | 0.05 | 0.08 | 0.08 | 0.08 | 0.08 | 0.06 | 0.01 | 0.04 | 0.06 | 0.05 | 0.05 | 0.05 | 0.02 | 0.02 | 0.01 | 0.00 |  |  |  |
| 36 | B. venatorum, LC005775 | 0.01 | 0.08 | 0.00 | 0.02 | 0.05 | 0.06 | 0.05 | 0.04 | 0.02 | 0.01 | 0.05 | 0.05 | 0.05 | 0.01 | 0.01 | 0.01 | 0.02 | 0.02 | 0.05 | 0.08 | 0.08 | 0.08 | 0.08 | 0.06 | 0.01 | 0.04 | 0.06 | 0.05 | 0.05 | 0.05 | 0.02 | 0.02 | 0.01 | 0.00 | 0.00 |  |  |
| 37 | Babesia sp. YZ-2012, JQ993427 | 0.02 | 0.09 | 0.02 | 0.00 | 0.05 | 0.06 | 0.05 | 0.04 | 0.03 | 0.02 | 0.05 | 0.05 | 0.05 | 0.02 | 0.02 | 0.02 | 0.02 | 0.02 | 0.05 | 0.08 | 0.09 | 0.09 | 0.09 | 0.06 | 0.02 | 0.04 | 0.06 | 0.05 | 0.05 | 0.05 | 0.00 | 0.01 | 0.03 | 0.02 | 0.02 | 0.02 |  |
| 38 | Theileria parva, HQ895985 | 0.07 | 0.07 | 0.07 | 0.07 | 0.09 | 0.08 | 0.09 | 0.08 | 0.08 | 0.07 | 0.09 | 0.09 | 0.08 | 0.07 | 0.07 | 0.07 | 0.07 | 0.07 | 0.08 | 0.06 | 0.07 | 0.07 | 0.07 | 0.08 | 0.07 | 0.08 | 0.08 | 0.09 | 0.09 | 0.08 | 0.07 | 0.07 | 0.08 | 0.07 | 0.07 | 0.07 | 0.07 |
