# Supplementary material for: Molecular Detection and Characterization of Zoonotic and Veterinary Pathogens in Ticks from Northeastern China
Source: Front Microbiol. 2016 Nov 29;7:1913. doi: 10.3389/fmicb.2016.01913 (PMC5126052; doi:10.3389/fmicb.2016.01913)
Supplement: Supplementary file 7 [file Table_6.DOCX]

**Supplementary Table 6.** Detection of *Babesia* spp. in ticks from northeastern China by nested PCR.

| Tick species | No. of ticks tested | Prevalence  (%, 95% CI) | Heilongjiang* | | | | | |  | Jilin | | | | |
| --- | --- | --- | --- | --- | --- | --- | --- | --- | --- | --- | --- | --- | --- | --- |
|  |  |  | Subtotal no. ticks tested | no. positive pools/no. pools | *B.v* (%,  95% CI) | *B.m* (%,  95% CI) | *B.d* (%,  95% CI) | Sequence variants (%, 95% CI) |  | Subtotal no. ticks tested | no. positive pools/no. pools | *B.v* (%,  95% CI) | *B.m* (%,  95% CI) | Sequence variants (%, 95% CI) |
| *D. nuttalli* | 253 | 0 | 47 | 0/5 | 0 | 0 | 0 | 0 |  | 206 | 0/16 | 0 | 0 | 0 |
| *D. silvarum* | 204 | 0 | 29 | 0/3 | 0 | 0 | 0 | 0 |  | 175 | 0/11 | 0 | 0 | 0 |
| *H. concinna* | 412 | 2.9 (1.5-5.1) | 412 | 10/28 | 0 | 0 |  | 2.9 (1.5-5.1) |  | 0 | 0 | 0 | 0 | 0 |
| *H. longicornis* | 390 | 1.4 (0.3-4.8) | 146 | 2/11 | 0 | 0 | 0.7 (0.1-3.4) | 0.7 (0.1-3.4) |  | 244 | 1/15 | 0 | 0 | 0.4 (0.1-1.9) |
| *I. persulcatus* | 1669 | 2.5 (1.7-3.6) | 1276 | 27/88 | 1.6 (1.0-2.4)^b^ | 0.4 (0.2-0.9) | 0.2 (0.1-0.5) | 0.2 (0.1-0.5) |  | 393 | 7/27 | 0.3 (0.1-1.2)^b^ | 1.1 (0.4-2.6) | 0.5 (0.1-1.7) |
| Total | 2928 | 1.8 (1.4-2.4) | 1910 | 39/135 | 1.0 (0.6-1.6)^a^ | 0.3 (0.1-0.7) | 0.2 (0.1-0.4) | 0.7 (0.4-1.2) |  | 1018 | 8/69 | 0.1 (0.1-0.5)^a^ | 0.4 (0.1-1.0) | 0.3 (0.1-0.8) |

**B.v, B. venatorum; B.m,* *B. microti; B.d, B. divergens;* Sequence variants included *Babesia* sp.hl-hlj178 (KU862306) detected in *H. longicorni*, *Babesia* sp.Ip-hlj179 (KU862305) detected in *I. persulcatus*, *Babesia* sp. hc-hlj212 (KU862304) detected in *H. concinna*, and *Babesia* sp.Ip-hlj238 (KU862303) detected in *I. persulcatus*.

*^a,b^*Significant difference was found between the prevalence in the two tick species (*p*<0.05), analyzed by the Fisher's exact test.
