# Supplementary material for: Molecular Detection and Characterization of Zoonotic and Veterinary Pathogens in Ticks from Northeastern China
Source: Front Microbiol. 2016 Nov 29;7:1913. doi: 10.3389/fmicb.2016.01913 (PMC5126052; doi:10.3389/fmicb.2016.01913)
Supplement: Supplementary file 8 [file Table_7.DOCX]

**Supplementary Table 7.** The evolutionary divergence between sequences of *Hepatozoon*.

|  | | 1 | 2 | 3 | 4 | 5 | 6 | 7 | 8 | 9 | 10 | 11 | 12 | 13 | 14 | 15 | 16 | 17 | 18 | 19 | 20 |
| --- | --- | --- | --- | --- | --- | --- | --- | --- | --- | --- | --- | --- | --- | --- | --- | --- | --- | --- | --- | --- | --- |
| 1 | **Hepatozoon sp. hlj-dn242, KX016029** |  |  |  |  |  |  |  |  |  |  |  |  |  |  |  |  |  |  |  |  |
| 2 | **Hepatozoon sp. hlj-Ip229, KX016028** | 0.01 |  |  |  |  |  |  |  |  |  |  |  |  |  |  |  |  |  |  |  |
| 3 | H. felis, AB771545 | 0.02 | 0.02 |  |  |  |  |  |  |  |  |  |  |  |  |  |  |  |  |  |  |
| 4 | H. felis, KF034779 | 0.02 | 0.02 | 0.00 |  |  |  |  |  |  |  |  |  |  |  |  |  |  |  |  |  |
| 5 | H. felis, HQ829444 | 0.02 | 0.02 | 0.00 | 0.00 |  |  |  |  |  |  |  |  |  |  |  |  |  |  |  |  |
| 6 | Hepatozoon sp. JM-6, FJ595132 | 0.01 | 0.01 | 0.01 | 0.01 | 0.01 |  |  |  |  |  |  |  |  |  |  |  |  |  |  |  |
| 7 | Hepatozoon sp. JM-7, FJ595133 | 0.01 | 0.00 | 0.02 | 0.02 | 0.02 | 0.01 |  |  |  |  |  |  |  |  |  |  |  |  |  |  |
| 8 | H. canis, FJ497011 | 0.05 | 0.06 | 0.04 | 0.04 | 0.04 | 0.05 | 0.06 |  |  |  |  |  |  |  |  |  |  |  |  |  |
| 9 | H. seychellensis, KF246565 | 0.05 | 0.05 | 0.04 | 0.04 | 0.04 | 0.05 | 0.06 | 0.08 |  |  |  |  |  |  |  |  |  |  |  |  |
| 10 | H. americanum, EU249992 | 0.04 | 0.05 | 0.03 | 0.03 | 0.03 | 0.04 | 0.05 | 0.07 | 0.07 |  |  |  |  |  |  |  |  |  |  |  |
| 11 | H. canis, AY150067 | 0.05 | 0.06 | 0.04 | 0.04 | 0.04 | 0.05 | 0.06 | 0.01 | 0.08 | 0.07 |  |  |  |  |  |  |  |  |  |  |
| 12 | H. canis, KT267961 | 0.05 | 0.06 | 0.04 | 0.04 | 0.04 | 0.05 | 0.06 | 0.01 | 0.08 | 0.07 | 0.00 |  |  |  |  |  |  |  |  |  |
| 13 | H. canis, KC138532 | 0.05 | 0.06 | 0.04 | 0.04 | 0.04 | 0.05 | 0.06 | 0.01 | 0.08 | 0.06 | 0.01 | 0.01 |  |  |  |  |  |  |  |  |
| 14 | Hepatozoon cf. clamatae, HQ224963 | 0.06 | 0.07 | 0.06 | 0.06 | 0.06 | 0.06 | 0.07 | 0.10 | 0.07 | 0.10 | 0.10 | 0.10 | 0.10 |  |  |  |  |  |  |  |
| 15 | H. domergue, KM234649 | 0.04 | 0.05 | 0.04 | 0.04 | 0.04 | 0.04 | 0.05 | 0.07 | 0.03 | 0.05 | 0.07 | 0.07 | 0.07 | 0.06 |  |  |  |  |  |  |
| 16 | H. fitzsimonsi, KR069084 | 0.05 | 0.05 | 0.04 | 0.04 | 0.04 | 0.04 | 0.05 | 0.07 | 0.04 | 0.06 | 0.07 | 0.07 | 0.07 | 0.06 | 0.03 |  |  |  |  |  |
| 17 | H. ixoxo, KX512803 | 0.06 | 0.06 | 0.06 | 0.06 | 0.06 | 0.05 | 0.06 | 0.09 | 0.07 | 0.08 | 0.09 | 0.09 | 0.09 | 0.02 | 0.06 | 0.06 |  |  |  |  |
| 18 | Hepatozoon sp. CS-2012, KF318171 | 0.02 | 0.02 | 0.01 | 0.01 | 0.01 | 0.01 | 0.02 | 0.05 | 0.06 | 0.04 | 0.05 | 0.05 | 0.05 | 0.07 | 0.05 | 0.05 | 0.06 |  |  |  |
| 19 | Hepatozoon sp. European, EF222257 | 0.03 | 0.03 | 0.02 | 0.02 | 0.02 | 0.02 | 0.03 | 0.05 | 0.06 | 0.05 | 0.05 | 0.05 | 0.06 | 0.07 | 0.05 | 0.05 | 0.06 | 0.02 |  |  |
| 20 | H. theileri, KP119773 | 0.05 | 0.06 | 0.05 | 0.05 | 0.05 | 0.05 | 0.06 | 0.09 | 0.06 | 0.08 | 0.09 | 0.09 | 0.09 | 0.03 | 0.05 | 0.06 | 0.02 | 0.06 | 0.06 |  |
| 21 | H. ursi, EU041718 | 0.04 | 0.05 | 0.03 | 0.03 | 0.03 | 0.04 | 0.05 | 0.06 | 0.06 | 0.05 | 0.06 | 0.06 | 0.06 | 0.08 | 0.06 | 0.06 | 0.07 | 0.04 | 0.04 | 0.07 |
