# Supplementary material for: Molecular Detection and Characterization of Zoonotic and Veterinary Pathogens in Ticks from Northeastern China
Source: Front Microbiol. 2016 Nov 29;7:1913. doi: 10.3389/fmicb.2016.01913 (PMC5126052; doi:10.3389/fmicb.2016.01913)
Supplement: Supplementary file 9 [file Table_8.DOCX]

**Supplementary Table 8.** Detection of *Hepatozoon* spp. in ticks from northeastern China by nested PCR.

| Tick species | No. of ticks tested | Prevalence (%, 95% CI) | Jilin* | | |  | Heilongjiang | | | | |
| --- | --- | --- | --- | --- | --- | --- | --- | --- | --- | --- | --- |
|  |  |  | Subtotal no. ticks tested | no. positive pools/ no. pools | H. dn242 (%) | H. Ip229 (%) |  | Subtotal no. ticks tested | no. positive pools/ no. pools | H. dn242 (%)* | H. Ip229 (%)* |
| *D. nuttalli* | 253 | 1.6 (0.5-3.8) | 206 | 1/16 | 0 | 0.5 (0.1-2.3) |  | 47 | 3/5 | 4.4 (0.9-14.5) | 1.9 (0.1-9.2) |
| *D. silvarum* | 204 | 3.1 (1.2-6.9) | 175 | 4/11 | 2.8 (1.0-7.0) | 0 |  | 29 | 1/3 | 3.3 (0.2-19.0) | 0 |
| *H. concinna* | 412 | 0.5 (0.1-1.6) | 0 | 0 | 0 | 0 |  | 412 | 2/28 | 0 | 0.5 (0.1-1.6) |
| *H. longicornis* | 390 | 1.1 (3.4-2.8) | 244 | 3/15 | 1.4 (0.4-3.9) | 0 |  | 146 | 1/11 | 0.7 (0.1-3.4) | 0 |
| *I. persulcatus* | 1669 | 1.9 (1.3-2.7) | 393 | 6/27 | 1.7 (0.7-3.6) | 0 |  | 1276 | 22/88 | 1.9 (1.2-2.8) | 0.1 (0.0-0.4) |
| Total | 2928 | 1.6 (1.2-2.2) | 1018 | 14/69 | 1.4 (0.8-2.4) | 0.1 (0.0-0.5) |  | 1910 | 29/135 | 1.4 (1.0-2.1) | 0.2 (0.1-0.5) |

*H.dn242, Hepatozoon sp. hlj-dn242 (GenBank access no. KX016029), which was detected in *D.nuttalli* in Helongjiang province*;* H.Ip229, Hepatozoon sp. hlj-Ip229 (GenBank access no. KX016028), which was detected in *I. persulcatus* in Helongjiang province.
